# Supplementary material for: Spatio-temporal dynamics of pathogenic variants associated with monogenic disorders reconstructed with ancient DNA
Source: PLoS One. 2022 Jun 24;17(6):e0269628. doi: 10.1371/journal.pone.0269628 (PMC9231702; doi:10.1371/journal.pone.0269628)
Supplement: Attachment — Submitted filename: Response_to_rviewers.docx [file pone.0269628.s003.docx]

To the Editors of *“PLOS ONE”*

*Dear Sirs,*

*Thank you for reviewing our manuscript and for letting us resubmit a revised version. We find all comments to be very helpful and constructive. We have taken up your suggestions, and the manuscript has been revised accordingly. Notably, we now clearly acknowledge that sample bias, inherent to all ancient DNA studies, might have affected our inferences on the temporal dynamics and geographic prevalence of the considered mutations. We have now also included supplementary tables that will allow interested researchers to recreate our analyses on the deposited ancient human genome-wide data. We believe that these changes have resulted in a much better version of the manuscript which we hope is more befitting for publication in PLOS ONE.*

*Sincerely,*

*Prof. Draga Toncheva*

Еditor`s comments:

3. We note that Figure 4 in your submission contain [map/satellite] images which may be copyrighted. All PLOS content is published under the Creative Commons Attribution License (CC BY 4.0), which means that the manuscript, images, and Supporting Information files will be freely available online, and any third party is permitted to access, download, copy, distribute, and use these materials in any way, even commercially, with proper attribution. For these reasons, we cannot publish previously copyrighted maps or satellite images created using proprietary data, such as Google software (Google Maps, Street View, and Earth). For more information, see our copyright guidelines: <http://journals.plos.org/plosone/s/licenses-and-copyright>.

***Our reply:*** *Figure 4 uses a very rudimentary world map that is not copyrighted.*

Reviewer #1: This is an interesting paper. May be 'at the discussion section ' papers dealing with the human passage between Asia-to Europe/ Africa to Europe.

***Our reply:*** *We have now added to references dealing with ancient migrations from Asia and from Africa to Europe that might have impacted our inferences in the Discussion part of the manuscript, Line 206*

It would be interesting to add at the discussion section 'FVL in Urartian back to 3000 BC' as a reference

***Our reply:*** *We have now referred to this study in the Discussion part of the manuscript, Line 184*

Reviewer #2: Thanks a lot for this interesting work. I just have my doubts with the proposed temporal dynamics of the traits. Since we still have only access to a limited number of ancient human genomes which are in some periods overrepresented I am wondering how much one can really infer already with the current dataset temporal dynamics. At least I would like to see a statement on the above mentioned limitations.

***Our reply:*** *Temporal as well as geographic discontinuity is inherent to all ancient DNA studies, and we recognize our inferences might be to a certain extend biased. Nevertheless, the considered variants show very differential dynamics indicating that these are at least to a degree independent of the temporal sampling bias. Still, we have modified the text to convey that uncertainty in the inferences about temporal dynamics (Line 31, Line 81, and Line 145). We state in the Discussion part that sample bias might have affected our inferences about the temporal dynamics and geographical prevalence. (Line 206-207)*

In addition I was missing a supplementary table presenting your findings on single individuum level. I could imagine that some colleagues would be interested to check whether there deposited ancient human remains carried a disease SNP. Thanks in advance for addressing these minor comments.

***Our reply:*** *We have now included as supplementary tables all the ancient samples considered (n=2729), their sex, estimated age, region they were sampled and the published articles they have been used in, Supplementary table S1. Also is included a table with all samples in which any of the considered pathogenic variants were established (n=230), Supplementary table S2.*
